# Supplementary figures and images for: Transcriptome profiling reveals the role of ZBTB38 knock-down in human neuroblastoma
Source: PeerJ. 2019 Jan 24;7:e6352. doi: 10.7717/peerj.6352 (PMC6348090; doi:10.7717/peerj.6352)

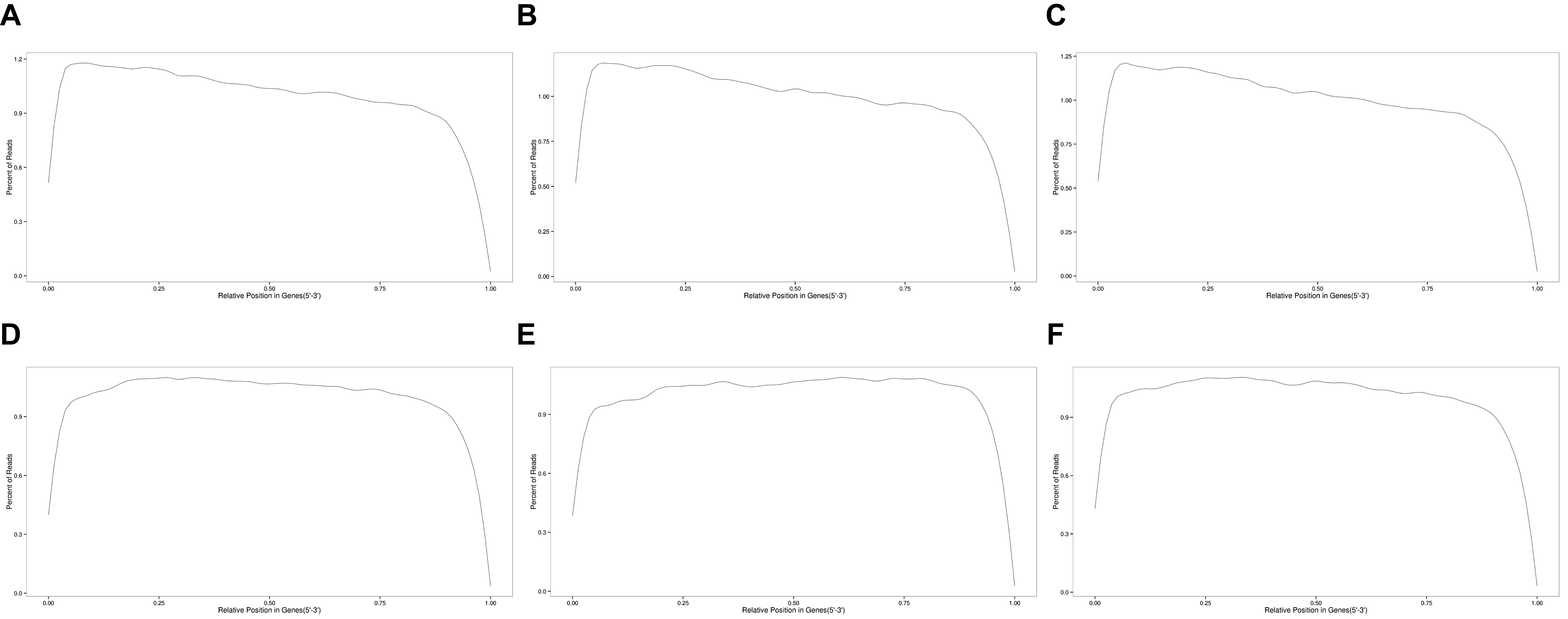

Supplement: Supplemental Information 1 [file peerj-07-6352-s001.zip › Supplementary material/Figure S1.png]

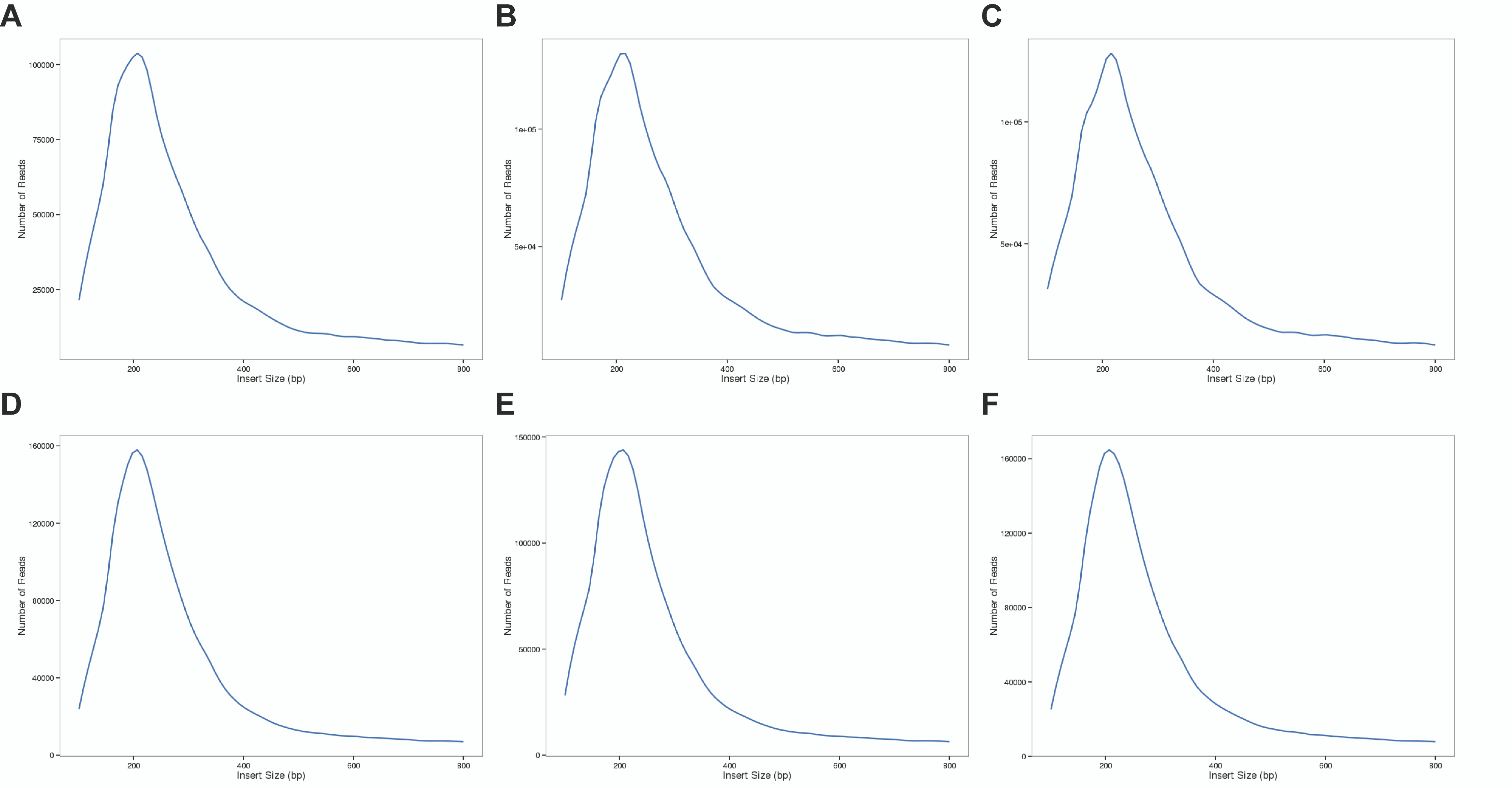

Supplement: Supplemental Information 1 [file peerj-07-6352-s001.zip › Supplementary material/Figure S2.png]

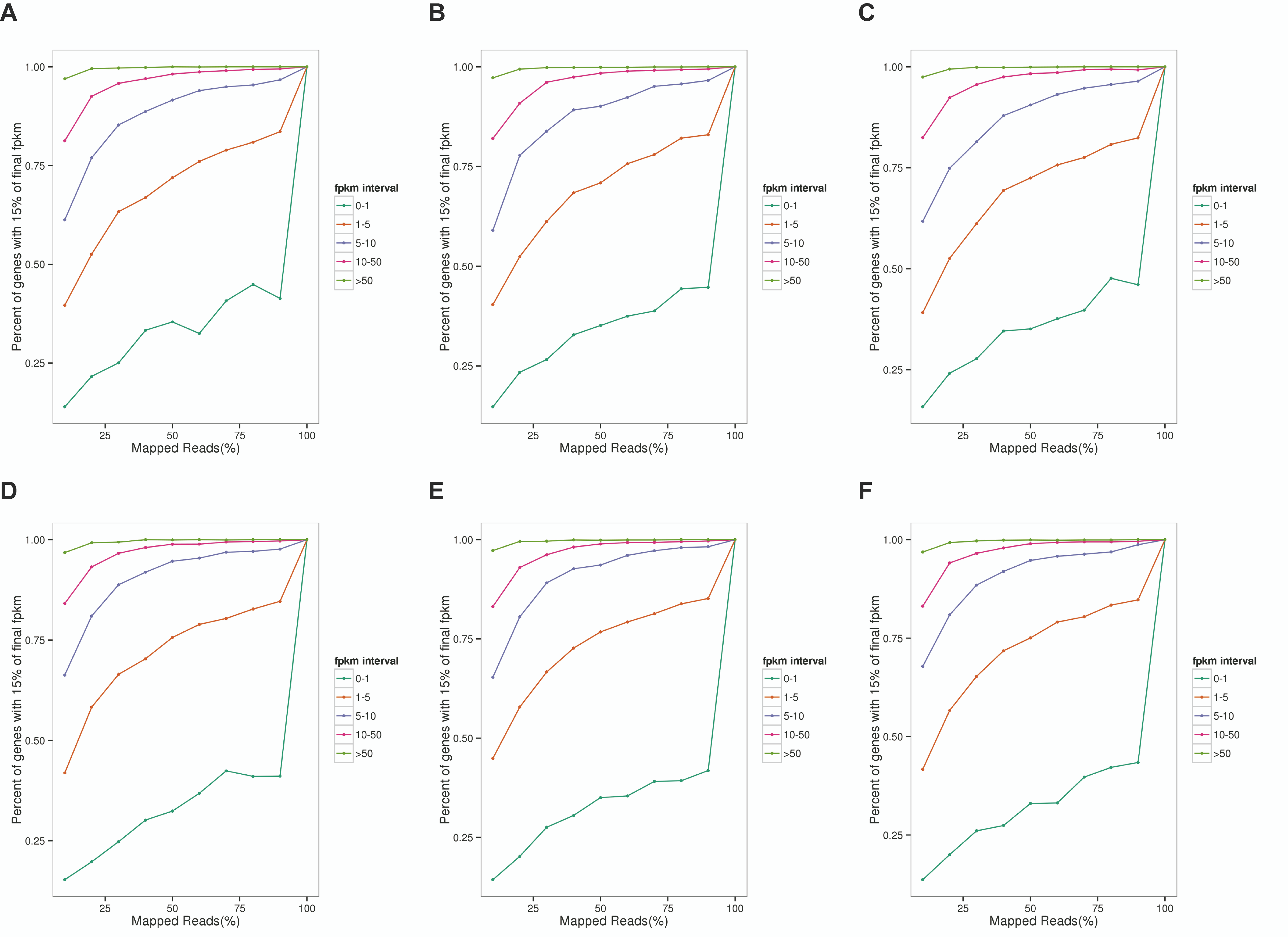

Supplement: Supplemental Information 1 [file peerj-07-6352-s001.zip › Supplementary material/Figure S3.png]

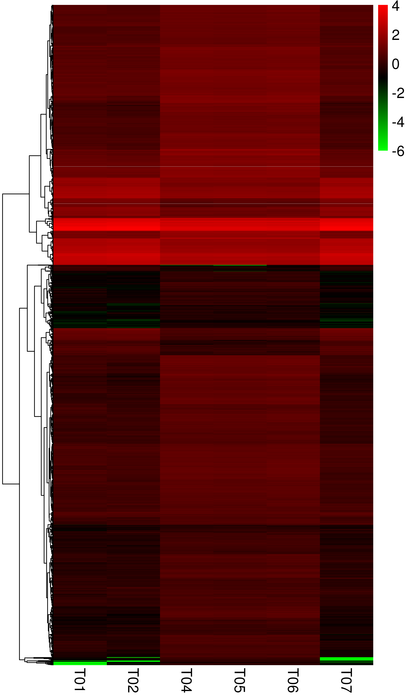

Supplement: Supplemental Information 1 [file peerj-07-6352-s001.zip › Supplementary material/Figure S4.png]

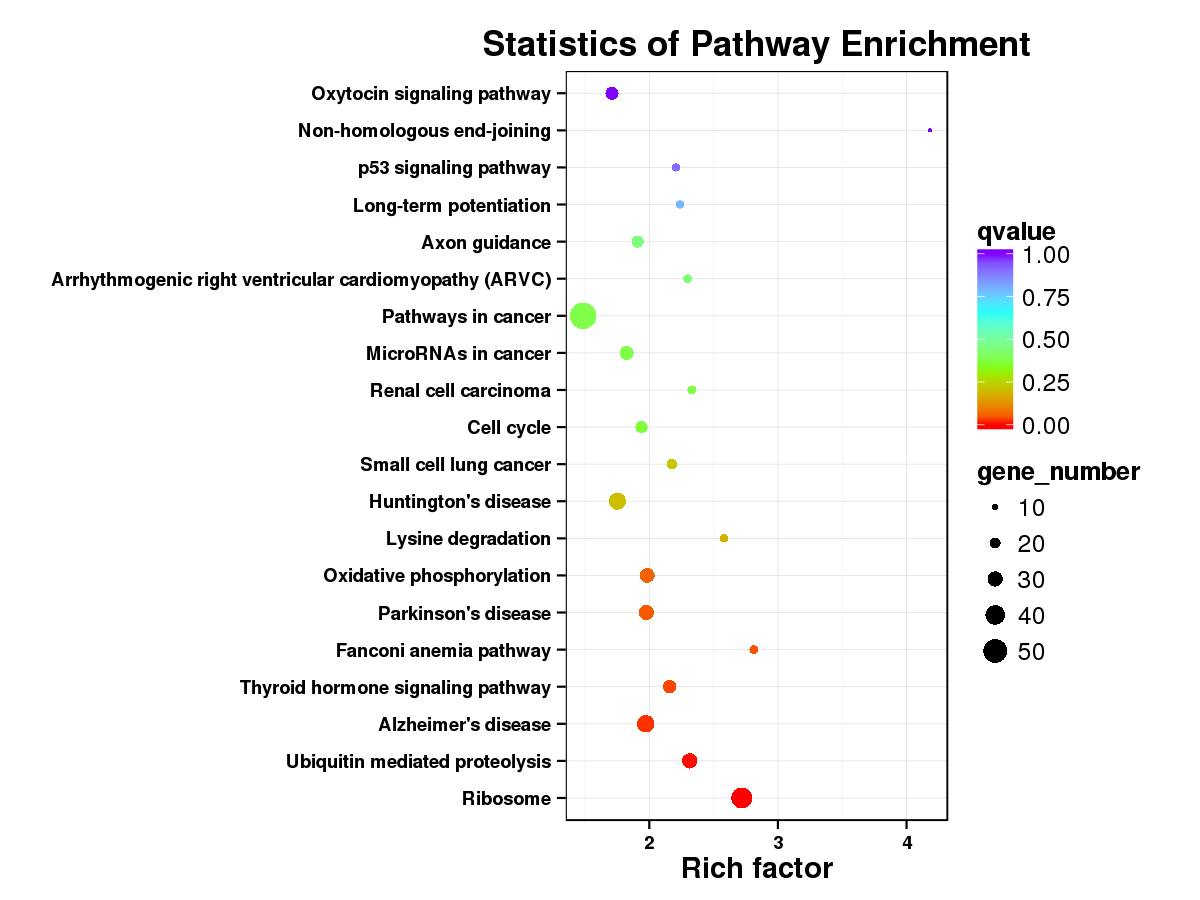

Supplement: Supplemental Information 1 [file peerj-07-6352-s001.zip › Supplementary material/Figure S5.png]

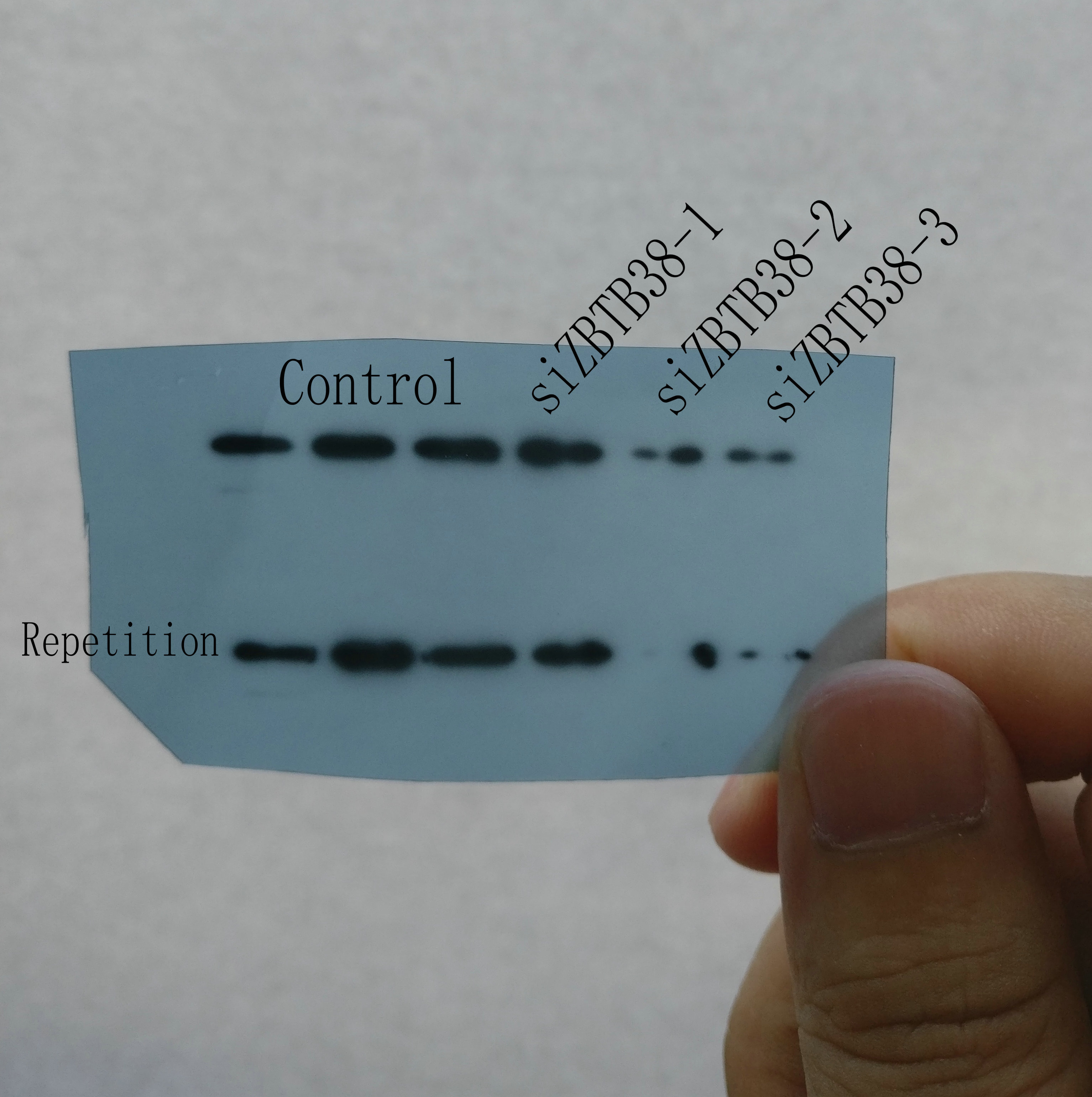

Supplement: Supplemental Information 2 [file peerj-07-6352-s002.jpg]
