# Supplementary material for: Transcriptome profiling reveals the role of ZBTB38 knock-down in human neuroblastoma
Source: PeerJ. 2019 Jan 24;7:e6352. doi: 10.7717/peerj.6352 (PMC6348090; doi:10.7717/peerj.6352)
Supplement: Supplemental Information 1 [file peerj-07-6352-s001.zip › Supplementary material/Supplementary figure legends.doc]

**Fig. S1 Location distribution of the mapped reads on mRNAs.** The X-axis indicated the normalized location of mRNAs, and the Y-axis indicated the percentage of reads fallen in the corresponding intervals to the total mapped reads. Owing to the different lengths of the reference mRNAs, when plotting, each mRNA was divided into 100 intervals according to its length, and the number and percentage of mapped reads in each interval were calculated. The proportions of mapped reads in all mRNA intervals were summarized in this figure. **A-C**:T01, T02, T07; **E-G**: T04, T05, T06.

**Fig. S2 Simulation of the inserted fragment length distribution.** The X-axis was the distance between the starting and ending sites of the alignment reads flanking the inserted fragments in the reference genome, with a range between 0 and 800bp, and Y-axis was the number of the flanking reads or inserted fragments with different distance between the starting and ending sites of the alignment. **A-C**:T01, T02, T07; **E-G**: T04, T05, T06.

**Fig. S3 Simulation of the saturation status of the transcriptome sequencing data.** Results in this figure represented the gene quantification analysis for 10%, 20%, 30, … and 90% of the total sequencing data randomly selected. The X-axis indicated the proportion of selected reads that mapped to the genome in the total mapped reads, and Y-axis indicated the percentage of genes with a difference in expression level of <15% in the selected sequencing data to each FPKM ranges. **A-C**:T01, T02, T07; **E-G**: T04, T05, T06.

**Fig. S4** **Cluster graph of differentially expressed genes** The abscissa represents the name and the clustering results of samples, while the ordinate represents the differential genes the clustering results of these genes. Different columns and different rows in the figure represent different samples and different genes, respectively. Besides, the color represents the level of gene expression [log2 (FPKM + 1)] in the sample. T01, T02, T07: the experimental group; T04, T05, T06: the control group.

**Fig. S5** **Scatter plot of the KEGG pathway enrichment analysis for DEGs.** Each circle in the graph represented a KEGG pathway, with the name of the pathway in the Y-axis and the enrichment factor in the X-axis. An enrichment factor was calculated as the ratio of the proportion of DEGs annotated to a given pathway in all DEGs to the proportion of all genes annotated to the same pathway in all genes. A greater enrichment factor indicted a more significant enrichment of the DEGs in a given pathway. The color of the circle represented the q-value which was the adjusted P after multiple hypothesis testing. A smaller q-value indicated a higher reliability of the significance of the enrichment of DEGs in a given pathway. The size of the circle indicated the number of genes enriched in the pathway, with bigger circle for more genes enriched. The top 20 pathways with the least significant Q value are presented.
